# A Proposed mHealth Intervention to Address Patient Barriers to Colposcopy Attendance: Qualitative Interview Study of Clinic Staff and Patient Perspectives

**DOI:** 10.2196/55043

**Published:** 2025-01-14

**Authors:** Jennifer R Hemler, Rachel B Wagner, Brittany Sullivan, Myneka Macenat, Erin K Tagai, Jazmarie L Vega, Enrique Hernandez, Suzanne M Miller, Kuang-Yi Wen, Charletta A Ayers, Mark H Einstein, Shawna V Hudson, Racquel E Kohler

**Affiliations:** 1 Department of Family Medicine and Community Health Rutgers Robert Wood Johnson Medical School New Brunswick, NJ United States; 2 Center Advancing Research and Evaluation for Person-Centered Care Rutgers Robert Wood Johnson Medical School New Brunswick, NJ United States; 3 Center for Cancer Health Equity Rutgers Cancer Institute New Brunswick, NJ United States; 4 School of Public Health Rutgers University Piscataway, NJ United States; 5 Cancer Prevention and Outcomes Data Support Rutgers Cancer Institute New Brunswick, NJ United States; 6 Cancer Prevention and Control Fox Chase Cancer Center Temple University Health System Philadelphia, PA United States; 7 Department of Medical Oncology Thomas Jefferson University Philadelphia, PA United States; 8 Department of Obstetrics, Gynecology and Reproductive Sciences Rutgers Robert Wood Johnson Medical School New Brunswick, NJ United States; 9 Department of Obstetrics & Gynecology and Women's Health Montefiore Medical Center Bronx, NY United States

**Keywords:** cervical cancer screening, colposcopy, HPV, human papillomavirus, mHealth, health communication, qualitative research, cancer screening, cancer, cervical cancer, screening, women, clinic staff, barrier, messaging, privacy, text message, qualitative, colposcopic, mhealth intervention, mobile phone

## Abstract

**Background:**

Cervical cancer disparities persist among minoritized women due to infrequent screening and poor follow-up. Structural and psychosocial barriers to following up with colposcopy are problematic for minoritized women. Evidence-based interventions using patient navigation and tailored telephone counseling, including the Tailored Communication for Cervical Cancer Risk (TC3), have modestly improved colposcopy attendance. However, the efficacious TC3 intervention is human resource-intense and could have greater reach if adapted for mobile health, which increases convenience and access to health information.

**Objective:**

This study aimed to describe feedback from clinic staff members involved in colposcopy processes and patients referred for colposcopy regarding adaptions to the TC3 phone-based intervention to text messaging, which addresses barriers among those referred for colposcopy after abnormal screening results.

**Methods:**

Semistructured depth qualitative interviews were conducted over Zoom [Zoom Communications, Inc] or telephone with a purposive sample of 22 clinic staff members (including clinicians and support staff members) and 34 patients referred for colposcopy from 3 academic obstetrics and gynecology (OB-GYN) clinics that serve predominantly low-income, minoritized patients in different urban locations in New Jersey and Pennsylvania. Participants were asked about colposcopy attendance barriers and perspectives on a proposed text message intervention to provide tailored education and support in the time between abnormal cervical screening and colposcopy. The analytic team discussed interviews, wrote summaries, and consensus-coded transcripts, analyzing output for emergent findings and crystallizing themes.

**Results:**

Clinic staff members and patients had mixed feelings about a text-only intervention. They overwhelmingly perceived a need to provide patients with appointment reminders and information about abnormal cervical screening results and colposcopy purpose and procedure. Both groups also thought messages emphasizing that human papillomavirus is common and cervical cancer can be prevented with follow-up could enhance attendance. However, some had concerns about the privacy of text messages and text fatigue. Both groups thought that talking to clinic staff members was needed in certain instances; they proposed connecting patients experiencing complex psychosocial or structural barriers to staff members for additional information, psychological support, and help with scheduling around work and finding childcare and transportation solutions. They also identified inadequate scheduling and reminder systems as barriers. From this feedback, we revised our text message content and intervention design, adding a health coaching component to support patients with complex barriers and concerns.

**Conclusions:**

Clinic staff members and patient perspectives are critical for designing appropriate and relevant interventions. These groups conveyed that text message-only interventions may be useful for patients with lesser barriers who may benefit from reminders, basic educational information, and scheduling support. However, multimodal interventions may be necessary for patients with complex barriers to colposcopy attendance, which we intend to evaluate in a subsequent trial.

## Introduction

Over the last few decades, the incidence of invasive cervical cancer in the United States has decreased due to the high uptake of preventive screening and access to human papillomavirus (HPV) vaccination [1-3]. However, there are large and persistent disparities in cervical cancer incidence [4,5] and mortality [6] among minoritized populations [7-11]. Importantly, cancer cases are higher among women living in socially deprived communities (eg, high proportions living below poverty) with high proportions of racial and ethnic minorities and historical racial segregation [4,12,13]. There is variation in screening by race and ethnicity, geography, and insurance [14-18], and low follow-up after an abnormal test result, especially for Black and Hispanic women [19-21]. Indeed, the majority of invasive cervical cancer cases are diagnosed among women who have never or rarely screened [8,22-24]. Guideline-concordant screening has decreased over the last 2 decades, leaving many women under-screened and at risk [25,26]. Poor follow-up behaviors and barriers have also been documented among women diagnosed with cancer [8,27].

Follow-up management for abnormal screening results among minoritized groups is influenced by a myriad of barriers. Patients’ low awareness or lack of knowledge [9,28-33], feelings of anxiety [29,33], and fear of receiving a fatal cancer diagnosis [31,34] are well-documented barriers to follow-up attendance. Patient confusion after receiving and discussing abnormal Papanicolaou or cotesting (ie, Papanicolaou and HPV tests) results with providers is common [35,36]. Delays in results delivery and challenges in appointment scheduling also contribute to low follow-up. Structural barriers such as lack of time, transportation, and childcare have also hindered colposcopy attendance [30]. Neighborhood factors, specifically living in more resource-deprived communities, and distance to care also influence time to follow-up, receipt of treatment, and ultimately outcomes, particularly among Black and Hispanic women [13,37-39]. Addressing the multiple, complex barriers to colposcopy remains critical to cervical cancer prevention.

Interventions using patient navigation [40,41] and tailored telephone counseling [42-44] have modestly improved colposcopy attendance. However, these human resource-intense interventions may have greater reach if they are adapted for new mobile health (mHealth) delivery platforms, which increase convenience and access to health information through automated direct communication [45,46]. Interventions using mHealth strategies have improved other aspects of cervical cancer prevention [47]. For example, online patient education tools administered before appointments increased knowledge about HPV or Papanicolaou testing and follow-up treatment [48,49]. In resource-limited settings, text messaging and app-based interventions were acceptable reminders [50], with some successfully increasing triage attendance [51,52] and others reducing psychosocial barriers to recommended follow-up [53-55]. However, many interventions have been developed outside of the United States and use one-way text messages to deliver cervical cancer screening results, which have not improved follow-up [54,55]. These studies highlight the need for additional features and content to address barriers and promote attendance. The flexibility [56,57] and ease of tailoring [58] interactive text messages have potential to increase an intervention’s impact, but acceptability and appropriateness must be evaluated for women referred for colposcopy.

Therefore, as part of a multisite effort to adapt an efficacious phone-based counseling intervention, Tailored Communication for Cervical Cancer Risk (TC3) [42,59], to be delivered through preprogrammed text messages [60,61], we sought to incorporate feedback from those engaged in colposcopy services—clinicians and support staff members—and patients who had been referred for colposcopy in clinics that serve minoritized populations. In this manuscript, we report the major findings from interviews where participants provided insight on critical barriers to colposcopy attendance and feedback on the proposed SMS text message intervention, useful functions the intervention should provide, and preferred delivery mode.

## Methods

### Intervention Adaptation

Our adapted intervention of the TC3 phone-based intervention, “Health Enhancement Resource System to address urban, cervical cancer disparities,” is a pilot SMS text message intervention designed to deliver tailored educational and counseling messages to women who were referred for colposcopy after receiving abnormal cervical screening results. In TC3, patients completed a telephone-based session with a trained health educator. Using a computer-assisted telephone interviewing system, the health educator asked the patient a series of questions related to five Cognitive-Social Health Information-Processing (C-SHIP)–informed topic areas: (1) knowledge and perceptions of the implications of positive HPV and abnormal Papanicolaou tests, cervical cancer, and colposcopy; (2) expectancies and beliefs concerning colposcopy; (3) interfering affect in relation to cancer and colposcopy; (4) values and goals related to follow-up and physical well-being; and (5) self-regulation in terms of overcoming and coping with potential barriers to colposcopy attendance, including structural barriers [43,62]. Based on patients’ responses, the computer-assisted telephone interviewing system algorithm gave the health educator a message to read to the patient for each C-SHIP barrier. In the Health Enhancement Resource System, patients awaiting colposcopy would receive a series of preprogrammed text messages adapted for text message delivery from TC3. For each domain, participants would receive a text asking them to assess their knowledge, level of concern, or perceived barrier, depending on the topic, on a 5-point scale. Participants would receive an intervention text message tailored to their response. For example, if expressing low knowledge or high concern about the barrier, they would receive an educational or counseling message; if expressing high knowledge or low concern about the barrier, they would receive an affirmation and move to the next text in the series. Participants could potentially receive all texts within 1 texting session or within a 2-week-long period before their colposcopy appointment.

### Study Design and Setting

Qualitative semistructured depth interviews developed within a constructivist approach [63] were used to discuss facilitators and barriers to colposcopy attendance and to elicit feedback about the fit of the adapted text message intervention with clinic staff members and patients. We developed different interview guides for clinic staff members and patients, including additional questions on the patient interview guide in relation to domains of the C-SHIP model (Multimedia Appendix 1). The objectives for both sets of interviews were to determine whether the adapted intervention aligned with patients’ needs and preferences and clinics’ communication and counseling processes. For this study, we recruited a purposive sample of clinic staff members, including physicians, and patients. We asked participants about their perceptions and either personal or known barriers to colposcopy attendance as well as their thoughts about what would be useful in a text-based intervention aiming to help patients attend their colposcopy appointments. We included questions about demographics and social characteristics. In addition, we asked patient participants pointed questions about intervention modality preference, frequency, and timing of receiving messages.

The study included 3 obstetrics and gynecology (OB-GYN) and colposcopy clinics affiliated with 2 universities that serve patients predominantly of low socioeconomic status and minoritized groups in different urban locations in New Jersey (Site A, clinics 1 and 2) and Pennsylvania (Site B, clinic 3). The 3 clinics within the 2 sites were selected with the intent to pilot the adapted text message intervention after receiving feedback from this interview study. The clinics have similar patient care management processes and perform similar rates of screening and follow-up care, including colposcopy.

### Participant Recruitment and Data Collection

Potential clinic staff member participants were referred to the study team by a clinic contact, who sent the team a list of clinic staff members involved in colposcopy services. At Site A, the clinics also sent the team a list of patients screened for eligibility and participation interest. Site B identified eligible patients through the electronic medical records, reviewing patients recently scheduled for colposcopy appointments. Clinic staff members were eligible to participate in interviews if they played a role in colposcopy services and were not a part of the research team. Patients were eligible if they were 18-70 years old, owned a cell phone with texting capability, and had received a colposcopy referral in the past year (completing the colposcopy was not a requirement). Patients were excluded if they were pregnant at the time of recruitment, had a history of malignancy, or had been diagnosed with invasive carcinoma of the cervix. Both groups of participants needed to be able to consent to participate. Site A participants were eligible if they spoke English or Spanish, whereas Site B participants were eligible if they spoke English.

A team of experienced health services researchers (JH, RB, BS, MM, JV, and Nicole Hernandez) trained in qualitative methods by a PhD health services researcher (JH and ET) and overseen by the larger team of cancer prevention and control, methodological, and clinical experts conducted qualitative semistructured depth interviews from July 2021 through March 2022 at Site A and until January 2023 at Site B. The team made up to 5 outreach attempts by phone and email to enroll potential participants into the study and emailed potential participants the study consent form to review ahead of the scheduled interview. Researchers answered all participant questions before asking participants for their consent. The trained researchers, all female and one bilingual in English and Spanish, conducted interviews in the participant’s preferred language. Interviews were held over the telephone or via Zoom.

We received contact information for 50 eligible clinic staff members (n=16 at Site A; n=34 at Site B) and 145 eligible patient participants (n=29 at Site A; n=116 at Site B). Across sites, we were unable to reach 17 clinic staff members and 61 patients within the prescribed number of attempts. A total of 39 patients declined and 4 dropped out of the study after providing consent. We were not able to schedule 4 clinic staff members and 7 patient interviews within the immediate time frame; saturation was achieved before being able to schedule these few interviews. Across sites, we interviewed 22 clinic staff members and 34 patients.

On average, clinician interviews were 53 minutes and patient interviews were 45 minutes. All interviews were recorded, professionally transcribed, translated from Spanish to English if necessary, and deidentified.

### Data Analysis

At each site, the same team of health services researchers who conducted the interviews met routinely to discuss interviews on a rolling basis, writing summaries and creating a matrix of participant responses to C-SHIP–informed barriers and perspectives on the content and mode of the proposed intervention. From these materials, we created a codebook of a priori and emergent topics. Interviews were concluded upon reaching saturation at each site, which was determined upon discussing interviews and finding that information aligned with previous interviews and did not offer substantially different perspectives. After this, the teams concretized the code book and coded in pairs using Atlas.ti software (Lumivero; version 9) to reach consensus upon code application. Each site’s analytic team discussed output and emergent themes with the larger study team, which included clinicians and health services researchers with expertise in cervical cancer prevention. The 2 sites compared and contrasted thematic findings, iterating themes until reaching crystallization [63]. The authors referred to the COREQ (Consolidated Criteria for Reporting Qualitative Research) [64] in reporting this research.

### Ethical Considerations

This study was approved by Rutgers University’s and Fox Chase Cancer Center’s institutional review boards (Pro2020002750 and 21-1080, respectively). All participants provided informed consent prior to being interviewed. Data were deidentified. Participants were given a US $30 gift card for taking part in the study.

## Results

### Sample Characteristics

The 22 clinic staff members interviewed across clinics and sites were similar (Table 1). Most were physicians, female, and non-Hispanic White. Clinic participants from Site A averaged 48 years of age; however, age was not assessed at Site B. Clinic staff members from both sites reported serving various roles related to colposcopy services; some physicians performed both screening (ie, HPV and Papanicolaou tests) and colposcopies while others primarily performed colposcopies in specialized clinics or settings (ie, colposcopy clinic and gynecologic oncology team). Support staff members typically scheduled colposcopies for patients, while physicians or advanced practice providers communicated with patients directly about their colposcopy results and the next steps thereafter.

The 34 patients interviewed across the clinics were similar in most regards. The majority were non-Hispanic Black, worked full-time, and were single, divorced, or separated. We noted variation among patients in terms of age and education across the clinics. At Site A, Clinic 1, participants averaged 46 years old and were more highly educated than the other 2 clinics. Clinic 2 participants were younger on average than the other 2 clinics, averaging 32 years old; these participants completed lower levels of education than the other 2 clinics. Site B, Clinic 3 participants averaged 41 years of age and varied across income and education (Table 2).

All patient participants, from both sites and the 3 clinics, had received an abnormal Papanicolaou test or positive HPV test result and were referred for colposcopy in the past year. Half were referred for their first colposcopy. All had smartphones and reported using their cell phones for multiple activities, like talking, texting, making appointments, using health and fitness and other apps, and using social media. While all reported text messaging as one of the main activities they did with their phone, their estimated phone time varied widely.

**Table 1:** Obstetrics and gynecology (OB-GYN) and colposcopy clinic staff members’ demographics by participating sites.

Characteristics		Site A			Site B		Total (N=22)
		Clinic 1 (n=8), n (%)	Clinic 2 (n=5), n (%)	Clinic 3 (n=9), n (%)			
**Race** **and** **ethnicity, n (%)**							
	Non-Hispanic Black	0 (0)	2 (40.0)	2 (22.2)		4 (18.2)	
	Non-Hispanic White	5 (62.5)	2 (40.0)	6 (66.7)		13 (59.1)	
	Non-Hispanic Asian	2 (25.0)	0 (0)	0 (0)		2 (9.1)	
	Hispanic	1 (12.5)	1 (20.0)	0 (0)		2 (9.1)	
	More than one race	0 (0)	0 (0)	1 (10.1)		1 (4.5)	
**Job title, n (%)**							
	Physician	5 (62.5)	3 (60.0)	7 (77.8)		15 (68.2)	
	Nurse	1 (12.5)	0 (0)	2 (22.2)		3 (13.6)	
	Advanced practice provider	1 (12.5)	1 (20.0)	0 (0)		2 (9.1)	
	Patient support	1 (12.5)	1 (20.0)	0 (0)		2 (9.1)	

**Table 2:** Demographic characteristics for patients referred for colposcopy at participating sites.

Characteristics		Site A			Site B		Total (N=34)
		Clinic 1 (n=9)	Clinic 2 (n=6)	Clinic 3 (n=19)			
Age (mean years, SD)		46.11 (15.01)	31.67 (7.39)	41.32 (10.06)		40.88 (11.88)	
**Race and ethnicity, n (%)**							
	Non-Hispanic Black	3 (33.3)	4 (66.6)	14 (73.7)		21 (61.8)	
	Non-Hispanic White	2 (22.2)	1 (16.7)	2 (10.5)		5 (14.7)	
	Non-Hispanic Asian	2 (22.2)	0 (0)	0 (0)		2 (5.9)	
	Hispanic	1 (11.1)	1 (16.7)	3 (18.8)		5 (14.7)	
	More than one race	1 (11.1)	0 (0)	0 (0)		1 (2.9)	
**Marital status, n (%)**							
	Single or never married	2 (22.2)	4 (66.6)	13 (68.4)		19 (55.9)	
	Married or living with partner	5 (55.6)	1 (16.7)	2 (10.5)		8 (23.5)	
	Divorced or separated or widowed	2 (22.2)	1 (16.7)	4 (21.1)		7 (20.6)	
**Employment status, n (%)**							
	Employed full-time	4 (44.4)	3 (50.0)	9 (47.4)		16 (47.0)	
	Employed part-time	0 (0)	1 (16.7)	4 (21.0)		5 (14.7)	
	Unemployed	1 (11.1)	2 (33.3)	4 (21.0)		7 (20.6)	
	Disabled	2 (22.2)	0 (0)	2 (10.5)		4 (11.8)	
	Retired	2 (22.2)	0 (0)	0 (0)		2 (5.9)	
**Annual income, n (%)**							
	US $15,000	0 (0)	0 (0)	6 (31.6)		6 (17.6)	
	US $15,001-US $30,000	0 (0)	4 (66.6)	6 (31.6)		10 (29.5)	
	>US $30,000	6 (66.7)	2 (33.3)	7 (36.8)		15 (44.1)	
	Don’t know or refused	3 (33.3)	0 (0)	0 (0)		3 (8.8)	
**Educational attainment, n (%)**							
	Less than a high-school diploma	0 (0)	0 (0)	2 (10.6)		2 (5.9)	
	High-school diploma or equivalent	0 (0)	5 (83.3)	8 (42.1)		13 (38.2)	
	Some college	0 (0)	0 (0)	4 (21.0)		4 (11.8)	
	Vocational school	0 (0)	0 (0)	3 (15.8)		3 (8.8)	
	Bachelor’s degree or higher	9 (100)	1 (16.7)	2 (10.6)		12 (35.3)	
Follow-up appointment is for patient’s first abnormal cervical test, n (%)		5 (55.6)	4 (66.6)	8 (42.1)		17 (50.0)	

### Qualitative Findings

Below, we highlight the critical feedback participants gave regarding the text-based intervention. Clinicians and patients, despite patients’ demographic differences across clinics, were remarkably similar in their perspectives on the barriers they thought women encounter in attending their colposcopy appointments and in their thoughts on our proposed intervention concept, including the types of messages we should include. They had mixed preferences for the mode by which they thought counseling and informational content should be delivered. We report these themes and nuances below.

#### Address Variable Colposcopy Attendance Barriers

Clinic staff members and patients posited that women who do not attend their colposcopy appointments experience variable socioemotional and structural barriers (Table 3). They thought fear of having a biopsy or being afraid of receiving cancerous results prevented some women from attending appointments but might compel other women to follow up. Many clinic staff members stated that they thought patients would attend appointments if they understood the importance of the procedure in preventing cervical cancer. However, most of the patients we interviewed attended their appointments without feeling knowledgeable or prepared; they explained that they attended appointments to lessen their fear and take care of their health. Patients and clinic staff members acknowledged that structural barriers were difficult for many women to overcome, especially inflexible or hourly work and lack of childcare, transportation, or inadequate health insurance. They noted that clinics not having evening or weekend appointment hours and needing patients to schedule appointments several months ahead likely increased the potential for patients not attending appointments. In addition, clinic staff members admitted their scheduling systems for making appointments were unnecessarily cumbersome, especially when patients needed to call back after receiving screening results rather than being able to schedule their colposcopy appointment during the same call. Patients thought being able to schedule through an automated online system, like a portal or website, would be more efficient and easier than having to call a scheduler, especially because they could use automated systems after clinic hours.

**Table 3:** Clinic and patient perspectives on common colposcopy attendance barriers.

Theme	Clinicians	Patients
Fear or avoidance	*Sometimes people are scared of the biopsies and sometimes they don’t think that it’s [the colposcopy] necessary*. [Site A, Clinic 1, Clinician, non-Hispanic Asian, 43 years old]	*They’re fearful that it could be cancer, so that’s why some people say, oh, just let it be. . . . They don’t want to discover that they have positive results.* [Site A, Clinic 1, non-Hispanic Asian, 66 years old]
Structural barriers	*They face a lot of challenges and a lot of struggles, just being able to get to their appointments . . . . They’re making sure the appointment is going to be covered with their insurance. . . or they don’t have insurance. . . . being able to take off of work to come to clinic.* [Site B, Clinic 3, Clinician, non-Hispanic White, age not assessed]	*The childcare, because I’m a single mother and then I can’t schedule it [at certain times]. . . . [In addition,] it takes me an hour to drive to the hospital.* [Site A, Clinic 2, non-Hispanic White, 28 years old]
Scheduling process	*Part of it is that our schedules book out a little bit far. Our colposcopy services, they’re only offered at certain time of the day… scheduling can be important to our patients, especially ones who work non-traditional schedules.* [Site B, Clinic 3, Clinician, non-Hispanic Black, age not assessed]	*I was literally on the phone with her for ten minutes . . . between my work schedule and then you can’t go there while you’re having your period. And then I was also going on vacation. So really? I think we bounced between – I don’t know – 10 different dates*. [Site A, Clinic 1, non-Hispanic Black, 40 years old]

#### Provide Practical Health Information and Supportive Messaging

All participants expressed a need for an intervention to provide pragmatic, educational, and counseling messages during the time between receipt of abnormal cervical screening results and colposcopy appointments to help patients overcome socioemotional and knowledge-based barriers to attendance. They thought sending appointment reminders through text was essential. They also suggested that sharing information with patients that emphasized prevention, prepared patients for the procedure, and reduced anxiety would be key to improving colposcopy attendance (Table 4).

**Table 4:** Clinic and patient perspectives on the types of messages intervention should include.

Theme	Clinic staff members	Patients
Provide patient appointment reminders	*I think our whole patient-reminder system, which is just a phone call with a message left, is probably not the most effective. . . . Text messages are better. . . . 99 percent of the other appointments I go to, I get a text message that says, “reply C to confirm.* [Site A, Clinic 1, Clinician, non-Hispanic White, 48 years old]	*It’s sometimes unclear about when to follow up or if it’s necessary . . . what I should do when I leave the doctor. . . . I think it helps just to – yeah, just like as a reminder and knowing more about what’s going on with your appointments.* [Site A, Clinic 1, Biracial, non-Hispanic Black and White, 27 years old]
Emphasize prevention	*But even if there was a short little thing … would help them to understand why they need to go because I find that so many of our patients . . . they, a lot of times, are like, “Oh, I don’t know. I just got a call that I have this appointment.* [Site B, Clinician, Biracial, non-Hispanic White and Asian, age not assessed]	*I don’t know whether “cancer” will scare people off [but] maybe if you have an abnormal Pap smear and then you need a colposcopy maybe you need to tell them. You need this because this is a test that allows us to detect whether you have the beginnings, right.* [Site A, Clinic 1, non-Hispanic Black, 63 years old]
Reinforce information and prepare patients	*[A]ny reinforcement is excellent. . . . [A]s soon as you leave the office, you realize that you’ve got a question that you wanted to ask. And this may be something that helps.* [Site A, Clinic 2, Clinician, non-Hispanic Asian, 58 years old]	*I had to look everything up on my own. . . . [S]ome people might not even know where to start, or they may be too nervous to look it up. So if it was right there or provided, it may be a little easier.* [Site B, Clinic 3, non-Hispanic Black, 32 years old]
Reduce anxiety and provide support	*They’ve been anxious about all of these questions at home [and] these [texts] can relieve that. . . . This is exactly what we counsel them in the clinic….* [Site A, Clinic 1, Clinician, non-Hispanic Asian, 38 years old]	*I was so afraid of that appointment. So having a way to calm my nerves, to make me feel all right about the procedure, that is 100% for me.* [Site B, Clinic 3, Hispanic, 44 years old]

#### Provide Patient Appointment Reminders

When first asked about a potential text message intervention, most participants, clinic staff members, and patients assumed that the texts would be reminder texts. While clinics used patient portals, none had a text messaging system and relied on phone calls and letters to patients that clinic staff felt were “not the most effective.” Clinic staff members thought text reminders could alert patients to schedule childcare, find transportation or make other arrangements that would help enable them to attend their appointments. In addition, some patients noted that reminders would be helpful for them to track follow-up needs. Some said they were confused about when, if, and what kind of follow-up appointment they needed. A text could help know “when to follow-up or if it’s necessary . . . what I should do when I leave the doctor. . . .”

#### Emphasize That Cervical Cancer Can Be Prevented

Clinic staff members and patients also agreed that focusing on prevention and early detection was critical to increasing colposcopy attendance. Multiple participants noted that receiving text messages that explained why colposcopy was needed and how the procedure would prevent cancer–“help them understand ‘why’ they need to go”–would increase the likelihood of making an appointment. Clinic staff members and patients expressed that the texts needed to strike a delicate balance, presenting an adequate amount of information regarding cancer risk without instilling fear or raising new concerns; as 1 patient said, “I don’t know whether [using the word] ‘cancer’ will scare people off…but maybe you need to tell them.” Clinic staff members also suggested that messages could be an opportunity to promote HPV vaccination for cancer prevention and should be included in the intervention.

#### Reinforce Information Given by the Clinic and Prepare Patients for the Procedure

Clinic staff members said that the proposed messaging aligned with patients’ frequently asked questions and what they tell patients. They thought redelivering this information was important, as they believed patients did not retain information about colposcopy when receiving their abnormal screening results: “any reinforcement is excellent.” Patients said that texts about what to expect at the visit, including how long the procedure would take, if they would experience pain, and if they would need assistance post procedure, would help patients better prepare for the visit. Clinic staff members and patients thought including postcolposcopy instructions, such as how long to avoid sex if a biopsy is taken and the potential for light spotting to occur, would be helpful in preparing patients. Some patients wanted to know what follow-up plans could entail; however, some clinic staff members thought the purpose of the texts should be to encourage women to attend their immediate appointment and thought that projecting too far into the future would be too complicated and case-specific to capture in a brief text.

#### Reduce Anxiety and Provide Support

Patients reported not receiving enough information when they received their abnormal screening results and said that having more information would have eased some of the anxiety they felt while waiting for their colposcopy. They wanted information from a trustworthy source, such as their health care provider’s office, instead of having to search online, and they wanted detailed information on types of HPV, HPV transmission, and potential consequences of their abnormal screening results. Younger women wanted information on fertility, especially. Clinic staff members acknowledged that patients have anxiety–“they’ve been anxious about all of these questions at home”–and that the texts may help address that anxiety: “this is exactly what we counsel them in the clinic.” However, while some clinic staff members thought explaining some of these topics over text might be too complex or not generalizable to everyone receiving the texts, they agreed that explaining how the HPV virus may lie dormant for years and therefore may not be a direct result of a recent infidelity would help reduce the stigma of sexually transmitted infections. Some patients thought the intervention should include messages offering encouraging words or affirmations, which would help reduce stress: “I was so afraid… having a way to calm my nerves… that is 100% for me.”

#### Enhance Text Messages With Additional Forms of Information or Support

While clinic staff members and patients responded positively to a patient-facing intervention that provided reminders, health information, and supportive messages, they had mixed feelings about a text-only intervention. Many in our sample preferred texts because they wanted “very direct,” “short, quick pieces of information”; they thought texts would help patients “know quickly what [they] needed to do” (Table 5). Others liked the idea of texts, but suggested texts be supplemented with infographics and diagrams or links to credible websites where more detailed information could be found. Both clinic staff members and patients thought texts should provide a phone number to a clinic contact in the event a patient had additional questions and suggested linking patients to additional counseling services or a support group should patients experience elevated concern or distress. Some clinic staff members noted that patients could call the clinic if they needed help with insurance or transportation services, as well; some clinics have personnel like social workers, patient navigators, or front desk staff members who can work with patients to help them find a way to attend their appointments.

Some participants raised concerns about text messages in terms of fatigue and privacy. Some patients wanted the ability to opt in or out of threads, and a few preferred to have information sent by email or accessible through an app so they could peruse the intervention information in one place at one time at their convenience. Younger patients worried that someone else might see a message notification containing sensitive information on their phone screen. Clinic staff members worried about HIPAA (Health Insurance Portability and Accountability Act) violations or privacy and access issues for patients who shared phones with relatives. Some staff members thought older patients would not be as receptive to texts as younger patients, but we found no evidence for this from older patients in our sample.

**Table 5:** Clinic and patient preferences for mode of message delivery.

Theme	Clinicians	Patients
Text messages	*I think texting is a really good way to get short, quick pieces of information communicated.* [Site A, Clinic 1, Clinician, Non-Hispanic White, 48 years old]	*Texts tend to be shorter so I guess I would choose text. It’s easier to read and then if I needed to do something, the message would be very direct so I would know quickly what I needed to do.* [Site B, Clinic 3, Non-Hispanic White, 37 years old]
Texts plus links to additional information	*Maybe text message ahead a link that you could click for information or also if you had any questions*. [Site A, Clinic 2, Support Staffs, Non-Hispanic White, 46 years old]	*Oh, that would be great, reliable information. . . . If there’s a link that can explain to you what’s happening and the reason why it’s happening, that would be so helpful.* [Site B, Clinic 3, Hispanic, 44 years old]
Texts plus a person who can counsel	*I think certain things might need clarification. I just find that when I call patients . . . I don’t know how that would translate through text. And I also wonder if it would feel a little impersonal as a patient to get like counseling through a text message.* [Site A, Clinic 1, Support Staff, Hispanic, 31 years old]	*There’s some situations that text messages is not going to work. . . . [If] you need some information, I can send you something on text messages. You can read about it. [But] If you have more doubts and you feel like you need some support, I have a counselor that can assist you. . . .* [Site A, Clinic 1, Hispanic, 33 years old]

## Discussion

### Principal Results

We conducted this study to elicit feedback from clinic staff members and patients with colposcopy experience. Despite differences in our sample between clinic locations, participant roles, and patient age and educational characteristics, participants overwhelmingly agreed that an intervention providing colposcopy appointment reminders, abnormal screening results and colposcopy procedure information, and psychosocial and structural barriers counseling is needed for patients awaiting colposcopy appointments. As noted in the literature and echoed throughout this study, patients may experience varied and often multiple barriers to attendance [30]. In our adapted SMS text message intervention, we proposed to assess and address C-SHIP–related [62] barriers by providing information and counseling texts related to barriers patients endorse. This intervention has the potential to address many of the patient needs participants discussed, which align with C-SHIP constructs of knowledge and perception, expectancies and beliefs, and interfering affect. In addition, although there was interest in a text message intervention, participants expressed that texts alone may not be sufficient for or desired by all patients. Participants conveyed that patients who want additional information or who need additional support in overcoming barriers should be linked to counselors or other resources to enhance the intervention’s potential to increase patient attendance.

mHealth strategies have been successful in improving prevention and health education among underserved minoritized populations [65], the population experiencing the greatest cervical cancer disparities. We heard from clinic staff members that health education is critical for follow-up attendance [66]. Our proposed text intervention is foundational for activated patients [67] who need basic information to assist in their decision-making about their colposcopy appointment. In our previous study, patients described being confused about the meaning of their screening results and having limited knowledge of HPV risk and colposcopy; seeking information on their own often increased anxiety and confusion [36]. In this study, clinic staff members reported that patients often do not absorb the information provided at the time of abnormal screening results delivery. Both patients and clinic staff members thought that messaging to reinforce the commonality of abnormal results but also the importance of colposcopy in cervical cancer prevention would be critical for patients seeking information to enhance their decision-making. Providing information of this kind may help reduce anxiety and increase knowledge, leading to increased colposcopy attendance for these types of patients. However, similar to other studies noting privacy concerns [68], the intervention messages will not contain patient information about their specific screening results.

Our findings also suggested a need for additional communication through other delivery modes for other kinds of patients. Texts may be limited in their ability to engage and support all patients and address all relevant topics. Participants acknowledged that patients with more complex or numerous psychosocial [69] or structural barriers [30] likely need additional support in overcoming their attendance barriers than text messages can provide. This group of patients would benefit from being connected to a point person at the practice or a professional counselor or support group, in addition to having the text message serve as a primer. Some clinic staff members noted that they can help connect patients to local resources for structural barriers like transportation or childcare or help navigate charity care or insurance needs. However, as staffing resources vary by clinic, this solution may not be scalable.

Importantly, the barrier experienced most by the patients we interviewed was scheduling, which is beyond the capacity of our proposed intervention. Work and childcare conflicts make scheduling difficult but having to schedule appointments months out aggravates these and other challenges especially pertinent to patients who have limited capacity to foresee or control their futures [18,70]. Clinic scheduling systems themselves were cumbersome for some patients. Including automated scheduling in conjunction with text reminders may help patients attend their follow-up appointments by reducing barriers to scheduling [71]. Patient-centered scheduling systems are essential structural elements that health systems can incorporate to improve appointment attendance in general and colposcopy appointments specifically.

### Future Research

Due to the strength and commonality of perspectives across clinic staff members and patient groups, and additional feasibility testing [61], the team expanded the intervention design to include brief health coaching sessions over the phone. Designing with efficiency and scalability in mind [72,73], this additional intervention component will be reserved for patients who experience complex barriers to attendance and missed appointments. Health coaching interventions, which are patient-centered and goal-focused, have improved health outcomes by empowering patients to self-manage their health or illness across multiple conditions and contexts [74-77]. The purpose of these calls will be to assess barriers, guide patients to existing resources, including connections to scheduling and structural barrier supports, and help them make a plan to attend the colposcopy appointment. The multimodal intervention will be tested this year.

### Limitations

This study had several limitations to consider. As mentioned above, we interviewed patients referred for colposcopy, many of whom had already attended a colposcopy appointment. The patients and staff members we interviewed spoke about others they knew and could imagine scenarios in which they themselves would experience such barriers [42,60,78]. The small number of patients we recruited who did not attend their appointments (n=5) noted similar barriers and provided similar feedback on how to improve the intervention. We anticipate health coaching professionals may help this group address attendance barriers. Also, we interviewed participants from 2 urban academic OB-GYN sites that have support services like care coordinators for their patients. As this was a purposive sample to gather perspectives on our proposed intervention, we found these perspectives helpful. However, our results may not be generalizable to clinics and practices with lower attendance rates and fewer supports for patients.

### Conclusions

Clinic staff members and patient perspectives are critical for designing appropriate and relevant interventions. These groups conveyed that text message-only interventions are useful for some patients but not for others. Multimodal interventions may be necessary for patients with complex barriers to colposcopy attendance, while patients with fewer barriers may benefit from reminders, basic educational information, and scheduling support.

## Appendix

Multimedia Appendix 1 Sample Interview Questions from Patient and Healthcare Staff Interview Guides.

## Abbreviations

C-SHIP: Cognitive Social Health Information Processing
COREQ: Consolidated Criteria for Reporting Qualitative Research
HIPAA: Health Insurance Portability and Accountability Act
HPV: human papillomavirus
mHealth: mobile health
OB-GYN: Obstetrics and gynecology
TC3: Tailored Communication for Cervical Cancer Risk
