# Supplementary material for: A Proposed mHealth Intervention to Address Patient Barriers to Colposcopy Attendance: Qualitative Interview Study of Clinic Staff and Patient Perspectives
Source: JMIR Form Res. 2025 Jan 14;9:e55043. doi: 10.2196/55043 (PMC11775484; doi:10.2196/55043)
Supplement: Multimedia Appendix 1 [file formative_v9i1e55043_app1.docx]

# Patient Interview Guide

# Need for text messaging intervention

Tell me about your cell phone:

How do you generally use your phone?

Do you currently use your phone for any health apps or messaging?

How do you think/feel about receiving counseling messages through texting?
 Probes:

- - Would you prefer receiving counseling messages through texting, voice mail, or email? And why?
  - Would adding messages on how to manage stress and negative emotions help increase your likelihood of coming to a follow up appointment?
  - How about including website links or videos for cervical cancer prevention?
  - Would you be interested in messages that provide information about the procedure colposcopy, too?

How often do you think you’d like to receive text messages about health counseling?

# C-SHIP-based Barriers

# Knowledge and Perception

# What is your understanding of what an abnormal Pap smear test result means?

When you learned that you had an abnormal Pap smear test result, what did you know about colposcopy?

**Expectancies and Beliefs**

After receiving the abnormal Pap smear test result, how important was it to you to follow-up on your results with any additional appointments or procedures your doctor may have recommended?

Did you discuss whether or not you should attend your colposcopy appointment with your family or friends?

**Affect**

# When you first learned about your Pap smear results and need for a colposcopy, how did you feel?

Probes:

# If they express they were worried/fearful/scared:

# What were you most worried/fearful/scared about?

- Were those feelings helpful or harmful in coming to clinic for a follow-up visit?
- What do you do to calm yourself? What helps?

If they do not express they were worried/fearful/scared:

- Were those feelings helpful or harmful in coming to clinic for a follow-up visit?
- Some people feel worried or fearful in these situations. How about you?
- What do you do to calm yourself when feeling stressed or worried or scared? What helps?

**Values and Goals**

What was the most important thing you considered in thinking about whether or not you should come to the clinic for colposcopy?

Probes:

- What might be possible benefits/advantages to you of receiving colposcopy or follow-up treatments or appointments?
- What concerns/disadvantages do you associate with receiving colposcopy or follow-up treatments or appointments?

The research shows that many women who have received abnormal test results do not go back to their doctor for their follow-up appointments or other procedures. Why do you think that is?

**Self-Regulatory Skills**

What would or could get in the way of you coming for colposcopy?

Did you have any problems or barriers that prevented you from following up with what your doctor recommended?

Probe:

- - How did you work around or overcome these problems or barriers?

What do you think would help other women come in for their colposcopy appointments?

# Healthcare Staff Message Evaluation Interview Guide

1. **Need for text messaging intervention**

Can you tell me about your role in colposcopy services (e.g., scheduling appointments, counseling patients, conducting exams/procedures)?

What is your perspective about colposcopy adherence in your clinic?

Why do you think some women with abnormal screening results do not want to attend colposcopy appointments?

Probes:

- - What types of emotional barriers/challenges do your patients face that prevent or hinder them in attending colposcopy appointments?
  - What types of structural or social barriers do you think they face that prevent or hinder their abilities to attend colposcopy appointments?

What have you noticed about patients and their lives that you think helps them attend colposcopy and follow-up appointments?

What do you do that you think helps patients attend colposcopy and follow-up appointments?

What do you think about the potential for text messages to help address patient barriers?

Probes:

- What do you think about their potential for improving adherence to recommendations?
- What kinds of messages do you think would help? What would messages need to be like to be successful?
  - What do you think about the potential of appointment reminder texts for improving adherence?

What do you think about having *counseling* text messages sent to patients needing colposcopy?

Do you have any concerns about sending counseling text messages?

1. **Evaluation of counseling messages**

These are the messages we may offer. Let’s review these messages and talk about them.

- - Do these messages make sense? Are they confusing or unclear?
  - Do you think these messages are appropriate in content and wording?
  - If not, how would you write them differently?
  - Do you think these messages would have helped your patients in dealing with problems coming in to the clinic for colposcopy and follow-up recommendations?
  - Is there anything missing?
  - Do you have any other comments or feedback?
